# Estrogens Induce Rapid Cytoskeleton Re-Organization in Human Dermal Fibroblasts via the Non-Classical Receptor GPR30

**DOI:** 10.1371/journal.pone.0120672

**Published:** 2015-03-17

**Authors:** Julie Carnesecchi, Marilyne Malbouyres, Richard de Mets, Martial Balland, Gallic Beauchef, Katell Vié, Christophe Chamot, Claire Lionnet, Florence Ruggiero, Jean-Marc Vanacker

**Affiliations:** 1 Institut de Génomique Fonctionnelle de Lyon, Université de Lyon, Université Lyon 1, CNRS UMR5242, Ecole Normale Supérieure de Lyon, Lyon, France; 2 Laboratoire interdisciplinaire de Physique UMR CNRS 5588, Université Joseph Fourier, Grenoble, France; 3 Laboratoires Clarins, Pontoise, France; 4 PLATIM, US8 / UMS3444 BioSciences Gerland, Lyon, France; II Università di Napoli, ITALY

## Abstract

The post-menopausal decrease in estrogen circulating levels results in rapid skin deterioration pointing out to a protective effect exerted by these hormones. The identity of the skin cell type responding to estrogens is unclear as are the cellular and molecular processes they elicit. Here, we reported that lack of estrogens induces rapid re-organization of the human dermal fibroblast cytoskeleton resulting in striking cell shape change. This morphological change was accompanied by a spatial re-organization of focal adhesion and a substantial reduction of their number as evidenced by vinculin and actin co-staining. Cell morphology and cytoskeleton organization was fully restored upon 17β-estradiol (E2) addition. Treatment with specific ER antagonists and cycloheximide respectively showed that the E2 acts independently of the classical Estrogen Receptors and that cell shape change is mediated by non-genomic mechanisms. E2 treatment resulted in a rapid and transient activation of ERK1/2 but not Src or PI3K. We show that human fibroblasts express the non-classical E2 receptor GPR30 and that its agonist G-1 phenocopies the effect of E2. Inhibiting GPR30 through treatment with the G-15 antagonist or specific shRNA impaired E2 effects. Altogether, our data reveal a novel mechanism by which estrogens act on skin fibroblast by regulating cell shape through the non-classical G protein-coupled receptor GPR30 and ERK1/2 activation.

## Introduction

Skin exhibits several functions providing protection against pathogens and ultraviolet irradiation, regulating hydration and temperature, exerting immunological surveillance and displaying endocrine activities. These functions are primary mediated by the epidermis, the most outer layer, whereas the underlying connective tissue layer, the dermis, represents the major mechanical component that protects skin against mechanical stress. The epidermis is highly cellular and is formed by several epidermal cell layers. In contrast, dermis contains sparse fibroblasts that are surrounded by an abundant extracellular matrix. Altered structure and reduced function of both epidermis and dermis are attributable to aging and result in skin deterioration, specifically in the face. This is characterized by dryness, atrophy, fragility, loss of elasticity, increased extensibility and wrinkling, as well as impaired wound healing. These undesirable aging effects are controlled by the genetic constitution of individuals (intrinsic aging) and are exacerbated by environmental factors (extrinsic aging) such as ultraviolet exposure and tobacco [1–2].

Several studies have shown that estrogens have beneficial and protective roles in skin biology [3–4]. Consistent with this view, reduced circulating levels of these hormones in post-menopausal women correlate with accelerated skin deterioration [4–5]. Conversely, estrogen supplementation in post-menopause women displays a beneficial role in skin, restoring dermal thickness and wound healing capacities [4, 6–11]. However these hormonal replacement strategies have been associated to an increased risk of developing breast and uterine cancer [11], preventing their use against skin aging. Little is known about the mechanisms by which estrogens protect skin from aging, despite the well-documented deleterious effects of hypoestrogenism on structure and function on the epidermis and dermis [2, 5, 11–12], and the strong correlation between skin collagen loss and estrogen deficiency resulting from menopause [4]. The identity of the skin cell type involved in estrogen protective effects is also unclear. Expression of estrogen receptor-corresponding mRNAs has been documented in dermal fibroblasts, the main producers of extracellular matrix proteins, including collagen. Nonetheless, the use of specific antibodies has shown that Estrogen Receptor (ER) α is mainly detected in sebocytes, whereas ERβ displays a broader expression in various skin cell types [13]. However, it should be noted that ER expression can vary according to skin location, with, for instance, higher receptor levels in facial- than breast skin [14]. Treatment with the selective estrogen receptor modulator raloxifen or, to a lesser extent, 17β-estradiol increased collagen biosynthesis in cultured human skin fibroblasts [15]. The molecular mechanisms by which estrogens act on collagen production in human dermis is not fully understood although studies have demonstrated a role of TGFβ, known to promote collagen production, in response to estrogens in human dermal fibroblasts [15–16]. However, besides changes in skin extracellular matrix content, the function of resident cells in the skin are likely influenced by estrogen. Although the role of exogenous estrogens in the integrity of human dermal fibroblasts has not been investigated, changes in fibroblast phenotype have been noted in aging skin [17–19].

Estrogens exert their actions via various molecular mechanisms. Genomic effects require ER α or ERβ, which are members of the nuclear receptor gene family [20]. These receptors directly bind to estrogen-response elements in the promoters of their target genes and regulate expression of the latters in a ligand-dependent manner. Such estrogenic effects eventually require translation of the regulated RNAs and can be thus blocked by the inhibitor of protein neosynthesis cycloheximide (CHX). Estrogens also exert so-called non-genomic effects, which depend on ER localized in the cytoplasm or at the plasma membrane [21–22]. In response to ligand, ER interacts with proteins such as PI3K or Src and induces secondary cascades that may culminate into the regulation of gene expression, although not via direct binding of ER to chromatin (reviewed in [23]). Since both genomic and non-genomic effects of estrogens depend on ER proteins, they can be blocked by ICI182,780, a synthetic compound that antagonizes estrogen binding to the receptors. A third mechanism of action has recently gained attention, which does not involve ER α or ERβ, and may not lead to regulation of gene expression. As such, these effects cannot be antagonized by CHX nor by ICI182,780 [24–26]. These effects are generally rapid (in the order of minutes) and can be mediated by binding of extracellular estrogens to the G protein-coupled receptor GPR30 (also called GPER for G Protein-coupled Estrogen Receptor), a seven-transmembrane receptor (reviewed in [27–28]). Depending on the cell system analyzed, E2-signaling through GPR30 may result in downstream activation of ERK1/2, EGFR, Src and/or PI3K [25–26, 29–31], leading to various cellular effects such as regulation of proliferation, cytoskeleton remodeling and migration. Interestingly, expression of GPR30 has been reported in the embryonic human skin fibroblast cell line WS1 suggesting the possibility of non-conventional actions of estrogen in skin [32].

To determine the direct, cell-autonomous effects of estrogens on human dermal fibroblasts, we have used isolated cells in primary culture. To avoid potential interference with photo-aging we chose cells originating from abdominal skin. Here we show that 17β-estradiol induces rapid cytoskeleton remodeling in isolated human dermal fibroblasts in primary cultures. This occurs through a GPR30-dependent, ER-independent pathway that lead to ERK1/2, but not Src/PI3K, activation.

## Materials and Methods

### Cells and reagents

Human dermal fibroblasts were provided by Biopredic (St-Grégoire, France) and originated from abdominal skin taken from female caucasian donors (aged between 28 and 41 years) with BMI <24 and no reported hormonal dysregulation. Cells were cultured in DMEM/HAM F-12 medium (Sigma) supplemented with 10% fetal calf serum (FCS) and 1% Zell Shield (Biovalley). MCF7 and MDA-MB231 cells were cultured in DMEM supplemented with 10% FCS and 1% Penicilline/Streptomycine. For desteroidation, serum was treated with activated charcoal (10g per 500ml; Sigma) for 4h and centrifuged. After three rows of treatment, serum was sterilized by filtration (0.22 μm). Phenol-red free medium was used for all experiments involving desteroidated serum. Proliferation was analyzed by cell counting at the indicated time.

17β-estradiol (E2), E2-BSA, ICI182,780 and cycloheximide (CHX) were purchased from Sigma. PD98059, G-1, LY294002, AG1478 were purchased from Cayman. G-15 was purchased from Tocris. All compounds were dissolved and stored according to the manufacturers’ instructions. All inhibitors (ICI182,780, CHX, PD98059, LY294002, AG1478, G-15) were added 1 h before E2 treatment. shGPR30 (TRCN0000235160, TRCN0000235161) and shControl (TurboGFP TRC2) were from Sigma.

### Wound-healing assays

For the wound-healing assays, cells were plated to confluence in a 12-well plate, and the cell surface was scratched using a pipette tip. Then, cells were treated or not with 10^-8^ M E2 and placed in the microscope round chamber (37°C and 5% CO2). The wound was imaged each 5 min for 48 h using a CCD CoolSNAP HQ monochrome camera mounted on a Timelapse Axiovert 100 M inverted microscope (10x objective) with a Metamorph software. Cells capacity to repopulate the scratched area was measured using ImageJ software. Four independent experiments were carried out.

### Transient transfections

1.5 10^4^ MCF7 cells were seeded in 96-well plates and transfected using Exgen500 following the manufacturer’s recommendations and 125 ng final DNA, comprising 12.5 ng ERE-Luc (generous gift from P. Balaguer, Montpellier, France), 25 ng ERα-encoding plasmid (generous gift from P. Balaguer, Montpellier, France) and pSG5 vector added as a carrier when needed. Transfection efficiency was normalized using βGal activity brought by cotransfection of CMV-βGal vector. For hDF transfection, 3 10^4^ cells were seeded in 24-well plates and transfected using Jet Prime following the manufacturer’s recommendations and 500 ng final DNA, comprising 100 ng ERE-Luc, 40 ng ER α-encoding plasmid and pSG5. Transfection efficiency was normalized using renilla luciferase activity brought by cotransfection of PRL vector (Promega). For shRNA transfection, 2,000 ng plasmid DNA were introduced in hDF cells.

### Immunostaining

For immunofluorescence experiments, cells (40% confluent) were cultured on glass slides, fixed with 4% paraformaldehyde and then washed with PBS 1X. For vinculin analysis, cells were seeded on glass slides coated with 0.5 μg of rat tail collagen I (Invitrogen) o/n at 4°C and blocked with 1% BSA for 1h30. Vinculin antibody (1/200, Sigma) diluted in PBS containing 1% BSA was used. Secondary antibody tagged with Alexa-488 (1/1000, Life Technology) and/or rhodamin-phalloidin (1/750, Sigma) were then added for 1h. Nuclei were counterstained with Hoescht staining. Immunofluorescence was analyzed using Zeiss-AxioImager or LSM780 Confocal microscope. Vinculin quantification was performed using computational analysis.

### Cell shape analysis

First, cells (30% confluent) were seeded on 6-well plates and cultivated into a room chamber (37°C and 5% CO2). Starvation was performed as described previously. Cell shape modification was followed by live imaging using Timelapse Axiovert. 4x4 images were taken by condition, every 2 minutes for 8 hours after estrogen treatment.

In a second time, cells area measurements were done on cells seeded on 6-well plates, at 40% of confluence. Starvation was performed as described previously. To avoid subjective observations, a blind treatment and/or blind observation were performed. Pictures were taken with Zeiss- Axiovert.

All images were processed and analysed with the open-source package, ImageJ with custom plug-in routines and PRISM Graphpad software.

### Protein analysis

For western blot analysis, cells were lysed in RIPA buffer or lysis buffer containing 1 mM NaVO_3_ and 10 mM NaF (for phosphoprotein analysis). All buffers were supplemented with Protease Inhibitor Cocktail (Sigma). Proteins (8–50 μg) were resolved on 10% SDS-PAGE, blotted onto PVDF membrane (GE-Healthcare) and probed with specific antibodies after saturation. For collagen extraction, cells treated for 4 days with E2 and ascorbic acid (50 μg/ml) were washed twice with PBS and directly lysed with NaCl/acetic acid buffer before pepsine digestion. After TCA extraction, pellets were resuspended in laemmli buffer and analyzed by western blot on 8% SDS-PAGE.

The antibodies (and their dilution) used in this study were: ERα (sc-8002 F-10, Santa Cruz, 1/1000), ERRα (GTX108166, Genetex, 1/2500), GPR30 (sc-48525-R, Santa Cruz, 1/500), total ERK (4695, Cell Signaling, 1/1000), p-ERK (4377, Cell Signaling, 1/2000), PI3K (4257, Cell Signaling, 1/800), pPI3K (4228, Cell Signaling, 1/500), Src (sc-8056 B-12, Santa Cruz, 1/1000), pSrc (2113, Cell Signaling, 1/800), hsp90 (API-SPA-830, Enzo Life Sciences, 1/1000), colI (20111, Novotech, 1/5000), colV (20511, Novotech, 1/2000), colVI (20611, Novotech, 1/3000), vinculin (1/600, Sigma).

### rt-qPCR

RNAs were isolated by Guanidinium-thiocyanate/phenol/chloroform extraction. 1 μg total RNA was converted to first-stand cDNA using SuperScript II retrotranscription kit (Invitrogen). Quantitative PCR were performed on 1% of the retrotranscribed mixture, using the sybr Green Jump Start Kit (Sigma Aldrich), 150 nM primers, in 96-well plates on a C1000 Thermal Cycler (BioRad). Oligonucleotide primers used in this study:

- 36b4: GTCACTGTGCCAGCCCAGAA and TCAATGGTGCCCCTGGAGAT
- ERα: CCGGCATTCTACAGGCCAAA and CCTTGGCAGATTCCATAGCCA
- ERβ: GGCCTCCATGATGATGTCCC and CGAACAGGCTGAGCTCCACA
- *COL1A1*: TTGCTCCCCAGCTGTCTTAT and AGACCACGAGGACCAGAGG
- *COL5A1*: CCGGATGTCGCTTACAGAGT and CTGCCTTTCTTGGCTTTCAC
- *COL6A1*: GGTATTCCAGGATGCAATGG and GGTATTCCAGGATGCAATGG
- *EGFR*: CCCAGTGACTGCTGCCACAA and CAGGTGGCACCAAAGCTGTA
- *CCND1*: CGTGGCCTCTAAGATGAAGGA and TCGGGCCGGATAGAGTTGT
- *CCND3*: TCACTGGCACTGAAGTGGAC and AGCTGGTCTGAGAGGCTTCC

### Statistical analysis

Data are represented as means±SEM of 4 donors. All statistical analysis were performed using One-Way Anova and Tukey’s test.

## Results

### 17β-estradiol regulates rapid cell shape change and focal adhesion re-organisation in human dermal fibroblasts

17β-estradiol (E2) is known to regulate cell proliferation in various tissues (e.g. breast, uterus) and cell lines in culture. We thus first analyzed whether proliferation of primary human dermal fibroblasts (hDF), isolated from abdominal skin of female donors was affected by this hormone. We did not observe any difference in the proliferation rate whether hDF were cultured in the presence of untreated- (10% fetal calf serum; FCS) or desteroidated serum (hereafter referred to as DS medium; S1a-e Fig.). To specifically evaluate the effects of E2, cells were cultured with DS supplemented or not with 10^-7^ M E2. As expected proliferation of MCF7 cells (a human estrogen-dependent breast cancer cell line) was clearly induced by E2 (S1f Fig.). In contrast, no significant effect of the hormone was observed on hDF originating from five different donors (S1a-e Fig). In addition cyclin D1 (but not cyclin D3) expression was transiently increased in MCF7 cells upon E2 exposure (as expected, [33] and references therein) reflecting the effect of the hormone on the G1-to-S transition (S1g Fig.). In contrast expression of neither cyclin D1 nor D3 was altered by E2 in hDF. Altogether this indicates that E2 does not regulate proliferation of hDF. For the experiments below we excluded cells from donor 3 which displayed a low proliferation rate.

**Fig 1:**
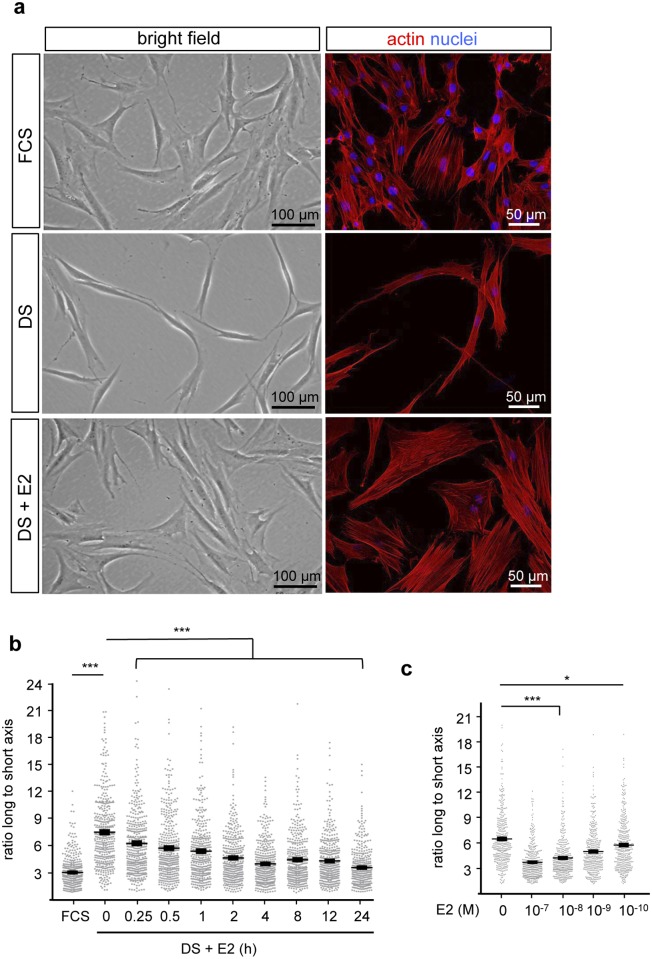
17β-estradiol remodels cell shape in dermal fibroblasts. **a**. hDF were cultured in the presence of untreated (FCS) or desteroided serum (DS) or in DS-containing medium supplemented with vehicle (DS) or 10–7M 17β-estradiol (DS+E2). Bright field microphotographs with cells from donor 1 are shown. **b-c**. Cells were cultured as indicated, analyzing kinetics of E2 exposure (**b**) or dose-response (**c**). Quantifications were performed indicating the ratio between the longest to shortest axis. Diagram represents mean±SEM of 400 cells quantified on 4 independent donors. 100 cells per condition were counted for each one donor. *p<0.05; ***p<0.001. Scale bars = 100 μm.

During our experiments, we observed a dramatic change in hDF cell shape according to the treatment. Indeed, as evidenced by bright field microphotographs, adherent cells cultured in the presence of FCS displayed a multi-angular shape (Fig. 1a). When cultured in the presence of DS, cells adopted a spindle-shaped morphology. This effect was completely reversed upon E2 addition (10^-7^ M; 24h) to the DS culture medium indicating that E2 is necessary and sufficient to promote multi-angular shape of hDF cells. To illustrate the rapidity and the magnitude of the effect of estrogen on cell shape, we performed live cell video-microscopy of 4X4 partial overlapping fields and create video mosaics. Videos showed that E2 treatment of hDF cultivated in DS media induces rapid and synchronous cell shape change (S1 Video) compared to untreated control (S2 Video). This effect was quantified by measuring the ratio between the length of the longer and shorter cell axes that as an indicator of spindle *vs* multi-angular cell shape. A highly significant cell elongation rate was observed when cells are cultured in DS medium compared to untreated media (FCS) (Fig. 1b). Consistent with the time-lapse video microscopy observations, the restoration of the long-to-short axis ratio upon E2 treatment was conspicuous as early as after 15 min of hormone exposure. Dose-dependent analysis indicated a highly significant effect (***p_value>0.0001) of E2 at concentrations above 10^-10^ M (4h treatment; Fig. 1c). We conclude that the rapid and synchronous cell shape change induced in DS medium is fully restored upon E2 treatment.

**Fig 2:**
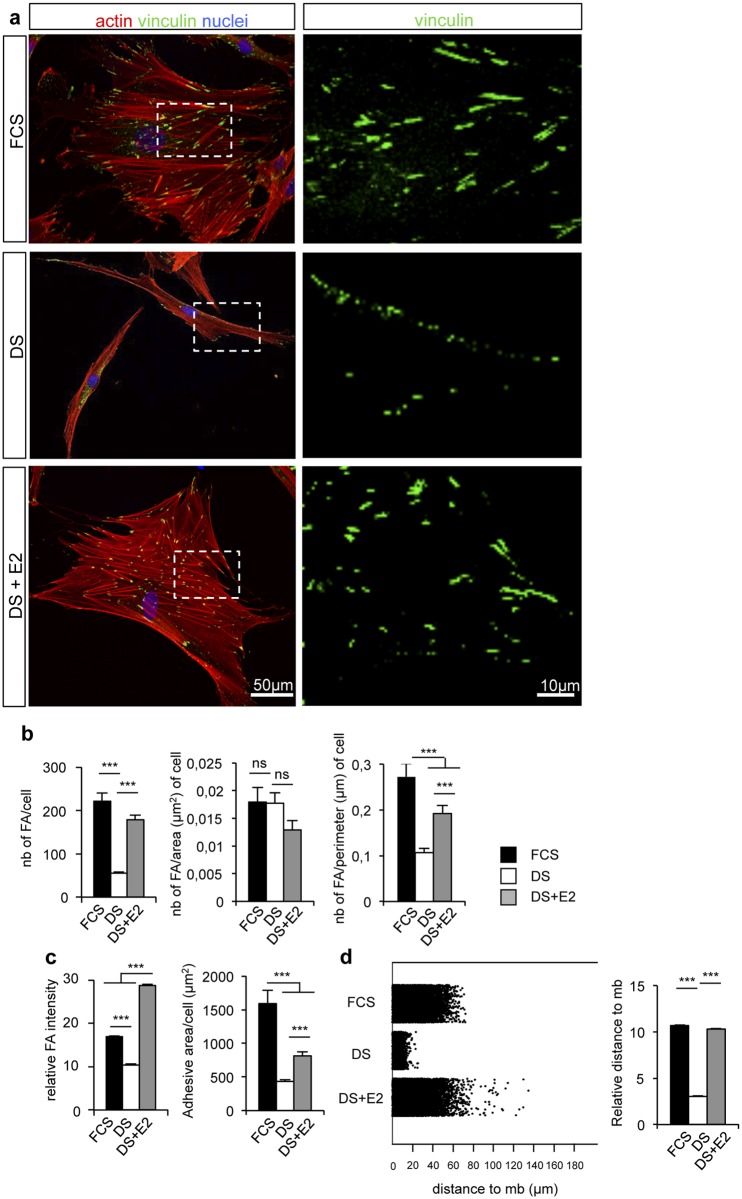
17β-estradiol treatment correlates with focal adhesion strengthening. **a.** Confocal pictures of donor 1 cultured in the presence of FCS, or DS supplemented or not with 10^-7^ M E2. Vinculin (green), actin (red) and nuclei (blue) were stained. **b.** Quantification of focal adhesions number, related or not to cell area or cell perimeter. **c.** Quantification of vinculin staining intensity and adhesive area by cell. **d.** Diagram of jitter plot (left) and mean (right) describing the distance of vinculin staining to the periphery of the membrane. Diagrams (except jitter plot) represent the mean±SEM of n>50 cells per condition. ***: p<0.001, ns: not significant.

Cell shape change is generally accompanied by actin cytoskeleton and focal adhesion (FA) remodeling [34–35]. We thus stained hDF with anti-vinculin antibody to visualize FA and with rhodamine-phalloidin to highlight actin fibers. While hDF cultivated in DS medium showed elongated shape with few FA sites mainly localized at the cell periphery and cell extremity, hDF cultured in the presence of E2 spread and displayed a multi-angular shape with multiple attachment sites similar to that observed with cells cultivated in presence of FCS (Fig. 2a). To quantify the number, size and distribution of vinculin-containing FA, morphometric analysis was conducted using image analysis software. The number of FAs that drastically decreased in DS cultured medium compared to FCS was significantly restored upon E2 treatment (Fig. 2b). Similar results were observed when the number of focal adhesions was normalized to cell perimeter. Morphometric analysis also revealed a significant loss of vinculin intensity and reduction of adhesive areas in DS medium as compared to FCS condition and to E2 treatment suggesting that hormone deficiency induces a loss of focal adhesion strength, a phenotype that can be fully restored by E2 addition (Fig. 2c). Interestingly, the FA intensity in cells treated with E2 was significantly higher than in the control conditions, indicative of a specific effect of E2 on adhesion strengthening rate. Because hDF cultivated in DS medium showed focal adhesion distribution at cell periphery and aligned along the cell long axis contrary to the apparent even distribution throughout the entire cell adhesion area observed in the two other conditions (Fig. 2a), we quantified their distribution by measuring FA distances to the cell membrane. In agreement with the immunofluorescence observations (Fig. 2a), a statistically significant change in focal adhesion distribution was observed in DS condition compared to FCS and E2 conditions (Fig. 2d). We conclude that restoration of cell shape change by E2 treatment is correlated with increased focal adhesion strength and a re-localization of attachment sites suggesting that E2 treatment is capable to restore focal adhesion number and distribution of hDF cultivated in DS media.

### 17β-estradiol treatment has no effect on cell migration and collagen synthesis rate

A relationship between focal adhesions and cell migrating capacity has been established [36]. We thus decided to examine the effect of E2 on hDF migration capacity by conducting scratch wound healing assays. Cell migration was evaluated by measuring the surface remaining uncovered by the cells at the end of the experiment. Although we did observe reduced cell migration in DS than in FCS conditions, supplementation with E2 did not rescue this phenotype indicating that the hormone is not sufficient to promote cell migration (S2a-b Fig.).

**Fig 3:**
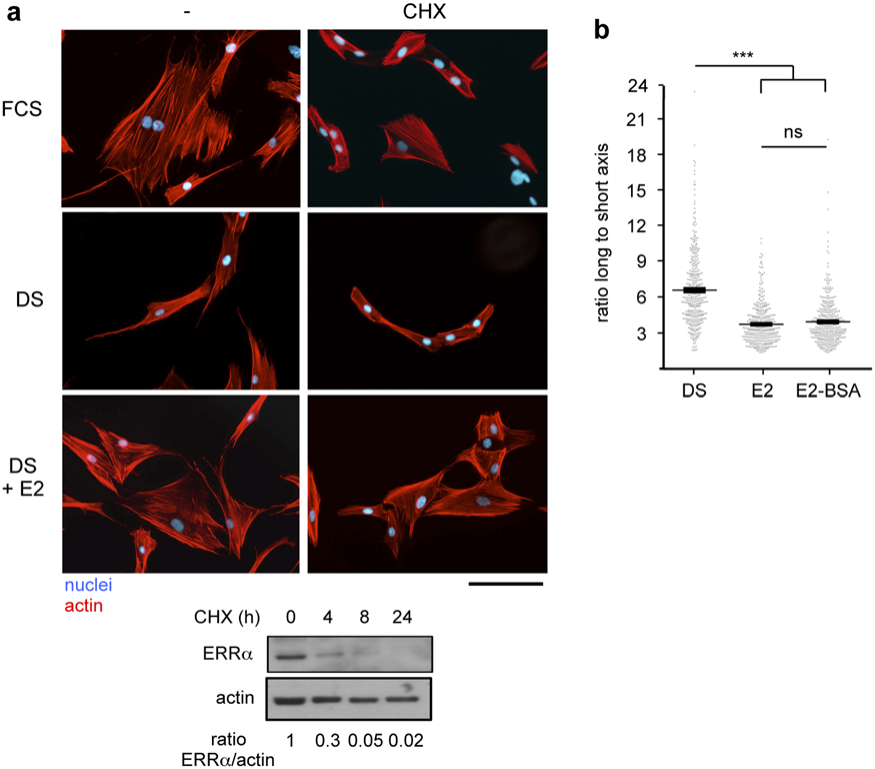
17β-estradiol effect on cytoskeleton re-organization involves non-genomic mechanisms. **a.** Cells were cultured in the presence of FCS, or DS supplemented or not with 10^-7^ M E2 and cycloheximide (CHX; 20 μg/ml). Actin and nuclei were stained. Scale bar = 100 μm. Lower panel: expression of the ERRα protein upon treatment with CHX for the indicated time was analysed by western blot with actin used as a loading control. **b.** Cells were cultured in the presence of DS for 2 days, then treated with 10^-8^ M E2 or its membrane-impermeable conjugate (E2-BSA). Cell shape was monitored as in Fig. 1b. n = 400 cells per condition. Values are mean +/- s.e.m; ns = not significant, ***p<0.001.

Estrogens have also been involved in the induction of collagen synthesis. We thus examined the effect of E2 on collagens I (colI), V (colV) and VI (colVI) that represent the major collagens found in dermis. However, in our conditions, neither E2 deprivation nor E2 treatment had a statistically significant effect on gene expression of all collagen type tested as measured by qPCR and of protein secretion as indicated by western blotting analysis (Fig. 3a-b).

### 17β-estradiol acts independently of conventional Estrogen Receptors

Rapid effects of E2 have been documented and do not involve target gene transcription nor protein neosynthesis. To evaluate the possibility that the effects of E2 observed on cytoskeleton organization are mediated by such non-genomic mechanisms, we treated hDF cells with E2 together with cycloheximide (CHX, an inhibitor of translation). We observed that CHX was unable to block cytoskeleton re-organization operated by E2 treatment (Fig. 3a). As a control, we verified that CHX treatment was efficient in blocking protein translation. The Estrogen Related Receptor α (ERR α) was reported to display a short half-life [37], the rapid decrease of the level of the unstable ERR α protein upon CHX treatment is therefore indicative of efficient translation inhibition. The fact that E2 acts on cell shape through non-genomic mechanisms suggests that the effects of the hormone may be mediated by membrane localized receptors. To examine this possibility we used the membrane impermeable E2-BSA conjugate. This compound was as efficient as E2 to reverse elongated cell shape (Fig. 3b) indicating that E2 acts at the cell membrane.

**Fig 4:**
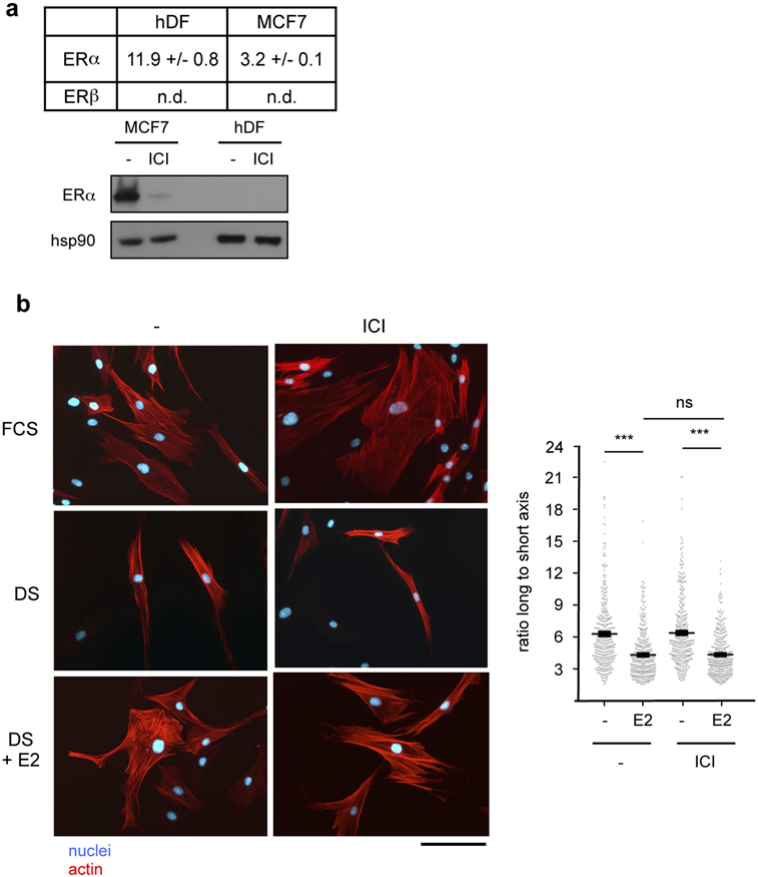
17β-estradiol effect on cytoskeleton re-organization involves ER-independent mechanisms. **a.** Expression of the indicated ER was determined in hDF and MCF7 cells. Upper panel, qPCR analysis- Expression of ERs is relative to the housekeeping gene 36b4 (values are in Ct of the indicated gene minus that of 36b4). Experiments were performed twice on the four donors each in triplicate. Values are mean +/- s.e.m. Lower panel, western blot analysis: ICI182,780 treatment (10^-7^ M) which induces proteasome-dependent degradation of ER [53] was used as a control. *HSP90* was used as a loading control. **b.** Cells were cultured as indicated. Actin and nuclei were stained. Scale bar = 100 μm. Right panel: cell shape was monitored as in Fig. 1b. n = 400 cells. ns = not significant; ***p<0.001.

To identify the receptors mediating the effects of E2 on hDF, we first investigated the expression of the conventional Estrogen Receptors (ERs, as members of the nuclear receptor superfamily). In hDF, ER (but not ERβ) was weakly detected at the mRNA level and not at the protein level, in contrast to MCF7 cells (Fig. 4a). We next examined whether this low ER expression was sufficient to mediate the effects of E2 on cytoskeletal reorganization. To this end, cells were treated with E2 (10^-8^ M) together with 10^-7^ M ICI182,780 (a specific ER antagonist). We observed that this compound was unable to block the effects of E2 on cytoskeleton organization changes (Fig. 4b). As a control we verified that ICI182,780 was efficient to penetrate hDF cells. To this end, hDF were transiently transfected with a plasmid encompassing estrogen-response elements controlling the expression of the luciferase reporter gene (ERE-Luc), together with ER. As expected reporter activity was increased upon E2 treatment, a phenomenon that was abrogated by ICI182,780 exposure indicating that this compound is an efficient ER antagonist in dermal fibroblasts (S4 Fig.). Taken together these results demonstrate that the conventional estrogen receptors do not mediate the effects of 17β-estradiol on cell shape change of dermal fibroblasts.

### 17β-estradiol induces rapid changes cytoskeleton organization in dermal fibroblasts through GPR30

**Fig 5:**
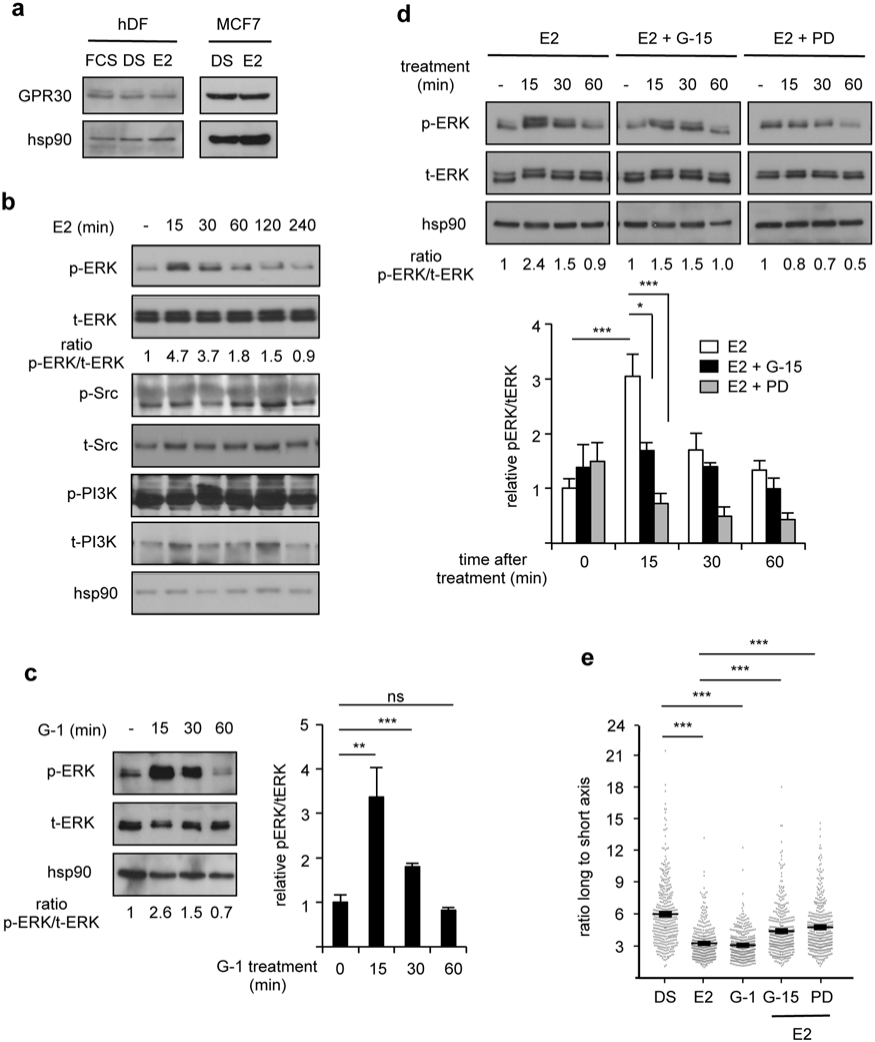
Pharmacological modulation of GPR30 alters hDF response to 17β-estradiol. **a.** Expression of the indicated proteins in hDF or MCF7. **b.** Expression of the indicated proteins upon E2 stimulation. Ratios of phosphorylated ERK (p-ERK) to total ERK (t-ERK) are indicated. **c.** ERK phosphorylation under the indicated conditions (G-1: GPR30 agonist). Right panel: quantification of the western blots performed on the four donors. Data are presented as mean +/- s.e.m. relative to vehicle-treated control. **d.** ERK phosphorylation under the indicated conditions (G-15: GPR30 antagonist; PD98059 [PD]: ERK inhibitor). Lower panel: quantification of the results as above. **e.** Cells shape monitored under the indicated conditions as in Fig. 1b. n = 400 cells. ns: not significant; *p<0.05; **p<0.01; ***p<0.001.

In several cell systems, GPR30 has been shown to act as a transmembrane estrogen receptor mediating rapid effects of the hormone. We thus investigated whether E2-induced cytoskeleton re-organization involves GPR30. We first showed that this protein is indeed expressed in hDF as well as in MCF7 cells, irrespective of the culture conditions (Fig. 5a). E2 signaling through GPR30 results in rapid and transient ERK1/2 activation [24–25, 30–31]. In agreement, ERK1/2 was phosphorylated within 15 min of E2 treatment (10^-8^ M) of hDF cells, and then progressively returned to basal situation (Fig. 5b). In contrast, no significant variation was observed in the phosphorylation levels of Src or PI3K.

**Fig 6:**
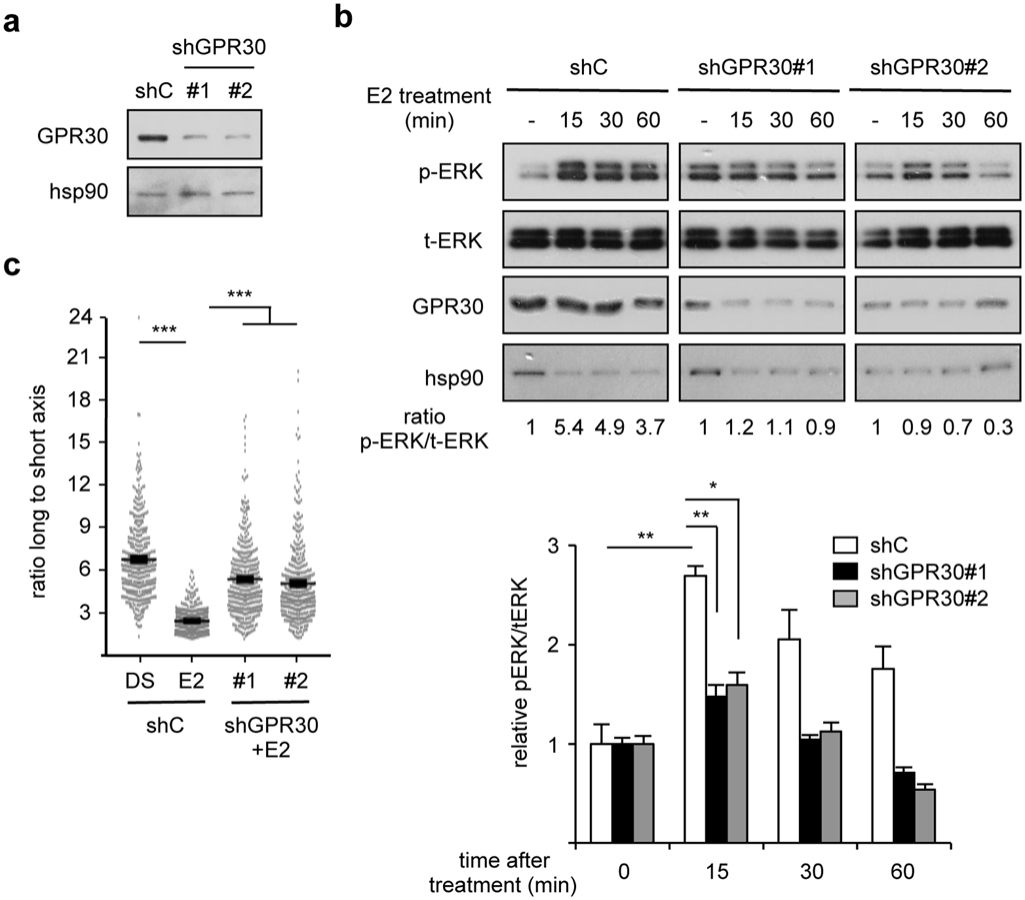
GPR30 mediates the effects of 17β-estradiol on hDF shape. **a.** Efficiency of shRNA vectors. hDF were transfected with shGPR30 (#1 and #2) or control (shC). Expression of the indicated proteins was determined 48 h after transfection. **b.** ERK phosphorylation under the indicated conditions. Ratios of phosphorylated ERK (p-ERK) to total ERK (t-ERK) are indicated. Lower panel: quantification of the western blots performed on the four donors. Data are presented as mean +/- s.e.m. relative to vehicle-treated control. **c.** Cells shape monitored under the indicated conditions as in Fig. 1b. n = 400 cells. ns: not significant; ***p<0.001.

If the activities of E2 are mediated by GPR30, then an E2-unrelated agonist of this receptor should phenocopy the effects of the hormone. In agreement with this reasoning we observed that treatment with 10^-8^ M G-1, a specific synthetic GPR30 agonist led to transient ERK1/2 phosphorylation (Fig. 5c), thus behaving similarly to E2. We next investigated whether inhibiting this receptor could reverse the effect of E2. To this end cells were treated with 10^-7^ M G-15, a specific synthetic GPR30 antagonist. We first observed that G-15 efficiently blocked the E2-induced ERK1/2 transient phosphorylation (Fig. 5d). Similar effects were also observed using 10^-5^ M PD98059, an inhibitor of ERK1/2 phosphorylation, as expected. In contrast, treatment with EGFR- (which is also expressed in hDF; S5a Fig.) or PI3K inhibitors (AG1478 and LY294002, respectively) did not abolished E2 effects on ERK1/2 phosphorylation (S5b Fig.). In addition, treatment with G-15 or PD98059, but not with AG1478 or LY294002, abolished the E2-induced cytoskeleton change (Fig. 5e and S5c Fig.). In agreement with a role for GPR30 in modulating cytoskeleton re-organization, 4h of treatment with G-1 resulted in similar effects to those displayed by E2 (Fig. 5e). Finally we examined whether GPR30 was required for the effects of E2. To this end, hDF were transiently transfected with GPR30-targeting shRNA, the efficiency of which is shown on Fig. 6a. After such a treatment, E2 was no longer capable of inducing ERK phosphorylation (Fig. 6b). Moreover the hormone was also unable to induce cytoskeleton changes upon GPR30 inactivation (Fig. 6c). Altogether our data show that 17β-estradiol induces cytoskeleton remodeling in human dermal fibroblasts in a GPR30- and ERK1/2-dependent manner.

## Discussion

In this report we show that estrogens induce actin cytoskeleton remodelling in isolated human dermal fibroblasts through an ER-independent, GPR30-dependent, non-genomic mechanism. In support of this statement, low amount of ER mRNA was expressed in hDF whereas no ERβ mRNA at all was detected in these cells. Furthermore ER protein was undetectable in western blotting experiments. This is in apparent contrast with previous reports that documented the expression of ERs in human skin [17, 38–40]. ERβ was the main ER expressed in fibroblasts whereas ER was expressed in sebocytes rather than fibroblasts [13]. However, it should be noted that these investigations used skin from forearm or scalp sometimes originating from post-menopausal women. In contrast we here used abdominal skin fibroblasts from pre-menopausal women. It is thus possible that ER expression varies according to hormonal status and/or anatomical origin of the fibroblasts.

In sharp contrast to ERs, GPR30, which has been proposed as a transmembrane estrogen receptor, is expressed at comparable levels in MCF7 and human dermal fibroblasts. This result is in agreement with a previous study showing the expression of GPR30, but not that of ER, in WS1 fibroblasts derived from embryonic human skin [32]. Interestingly the estrogenic effects that we observed in hDF were not blocked by the inhibitor of protein neosynthesis CHX (indicating that the hormonal effect does not require regulation of gene expression) nor by the specific ER antagonist ICI182,780 (indicating that the ERs are not involved in the process). These results are consistent with several reports indicating that E2 activity via GPR30 is not antagonized by ICI182,780 [24–26, 41]. In contrast, the synthetic GPR30 antagonist G-15 inhibited estrogen-induced ERK1/2 phosphorylation as well as actin remodeling. Moreover exposure to G-1, a specific GPR30 agonist mimicked the effects of estrogens treatment on ERK1/2 activation and cytoskeleton remodeling. In agreement coupling E2 to BSA, which renders the hormone unable to cross the cell membrane, does not impair its capacity to induce actin remodeling, indicating a membrane-based effect. Activation of ERK1/2 signaling cascade by E2 is consistent with a GPR30-mediated cascade [25, 30–31]. Furthermore the rapid ERK1/2 phosphorylation induced by estrogen treatment is also consistent with the minute range non-genomic actions of the hormone, as are the cytoskeleton re-organization after hormone exposure.

Estrogens have been reported to regulate cell morphology and to drive cytoskeleton re-organisation of several cell types as glial cells [42], carcinoma cell lines [43–44], endothelial cells [45–46], osteoblasts **[** 47], neurons [48], and oligodendrocytes [49]. Estrogen-driven re-organisation of actin cytoskeleton has been implicated in different aspects of cell biology such as cell migration or protection of cell integrity. Here we showed that estrogens induce rapid non-genomic re-organisation of actin cytoskeleton and focal adhesions and thereby restore the spread shape of human dermal fibroblasts cultivated in desteroidated medium. Adhesive area strongly modulated adhesion strength [34]. Cell-adhesion strength was shown to correlate with the size, length and distribution of focal adhesions and staining intensity of vinculin-containing FA [34–36]. We showed that fibroblast cultivated in desteroided medium displayed a reduction of FA number and intensity and reduced adhesive area both suggestive of a decreased adhesion strength, a phenotype fully restored by E2 exposure. E2 is thus necessary and sufficient to preserve cell sreading and actin cytoskeletal structures of hDF and thereby cell adhesive strength. It has been observed that, in aging skin, fibroblast can collapse due to a disruption of the extracellular matrix by MMPs [19]. Hormonal deficiency could be in part responsible for a reduction of cell anchoring capacities, resulting in fibroblast collapse. Importantly, our data defines a potential protective effect of sex hormone on fibroblast adhesive strength.

Intriguingly, cell migration capacities are reduced when dermal fibroblasts were cultured in DS-containing medium compared to untreated serum, an effect which seems not be rescued by E2 supplementation. This indicates that desteroidation of the serum also removes factors that are necessary for migration and are distinct from E2. This hormone is thus not sufficient alone to induce hDF migration although it is unclear whether it is necessary or not. On another hand, E2-induced, transcription-independent, actin remodeling and increased cell migration have been shown to occur in endothelial and endometrial cells [45, 50–51]. These effects apparently involve Src and PI3K activation and depend on a membrane localized ER protein, although a potential role for GPR30 has not been addressed. Taken together with our observation showing that Src and PI3K are not activated by E2 in human dermal fibroblasts, this may also suggest that E2 signaling through GPR30 is sufficient for actin remodeling whereas activation of cell migration could require additional cascades depending on a membrane localized ER signaling to Src/PI3K.

Our data show that cytoskeleton re-organization in dermal fibroblasts does not involve EGFR signaling which has however been reported to contribute to GPR30-dependent estrogenic effect in some cell types [24, 26, 30–31]. Although EGFR is expressed (at least at the mRNA level) in dermal fibroblasts, it is possible that additional factors are required for an E2-activation of this pathway. Whereas ERK1/2 phosphorylation is clearly required for E2-induced cytoskeleton remodeling, the identification of the effectors downstream of these kinases will require further investigations.

A bulk of evidence supports the effect of estrogens in collagen production. Nevertheless, we were not able to show any induction of expression of collagen type I nor of other collagen types such as collagen V and VI in cultured human dermal fibroblasts upon E2 treatment. It is possible that the effect of E2 on collagen synthesis is mediated by ERs rather than by GPR30. Our data provide information on the effects of E2 on isolated fibroblasts in primary culture. It remains possible that this hormone exerts distinct effects on dermal fibroblasts in vivo or even when they are cultured in the presence of keratinocytes. For example, one may hypothesize that keratinocytes could secrete factors that activate ER expression in fibroblasts, thereby modifying the nature of direct E2 signaling in fibroblasts. Alternatively, E2 may directly signal to keratinocytes, which would in turn secrete factors regulating fibroblast homeostasis. Such cell non-autonomous effects of E2 have indeed been described in the liver in which activation of ER signaling induces IGF-1 secretion that contribute to the progression of the estrous cycle [52].

Altogether our results point to previously unreported effects of E2 on human dermal fibroblasts. Our data could allow the establishment of easy tests to monitor the effects of estrogenic-mimicking compounds that could reverse the effects of menopause on skin. Hormone replacement therapies, aiming at restoring the pre-menopausal circulating levels of estrogens have been previously used to counteract the deleterious effects of menopause but have however been associated with an increased risk in breast cancer, in a manner that depends on the classical estrogen receptors. Our present data show that the effects of E2 on human dermal fibroblasts do not depend on these receptors and have thus a different mechanistic basis. Targeting GPR30 in skin, instead of the ERs, may thus circumvent the unwanted side effects of hormone replacement therapies.

## Supporting Information

S1 Fig 17β-estradiol does not regulate proliferation of hDF. Cells from donors 1 to 5 (**a** to **e**, respectively) were cultured in the presence of untreated (FCS) *vs* desteroidated serum (DS) (left panels) or in DS-containing medium supplemented with vehicle (DS) or 10^-7^ M 17β-estradiol (DS+E2) (right panels). **f.** MCF7 cells were cultured in DS-containing medium supplemented or not with 10^-7^ M 17β-estradiol. Proliferation is expressed relative to 0h. Values represent a single experiment performed in duplicate on single donors (two experiments performed per donor with similar results) with error bar representing S.D. Significance was estimated using one way ANOVA test. ***: *p*<0.001. Donor 3 displayed a low proliferation rate and was excluded from further analyses. **g.** Expression of cyclin D1 or D3 (CCND1 and CCND3, respectively) in hDF or MCF7 cells under the indicated conditions, determined by qPCR. Results are expressed relative to the expression of the 36b4 housekeeping gene. Experiments were performed on each for donors in triplicate. Values are mean+/-s.e.m. ns = not significant, ***: *p*<0.001.
(TIF)

S2 Fig 17β-estradiol does not regulate hDF migration. **a.** hDF were cultured in the presence of untreated (FCS) or desteroidated serum (DS) for 2 days, then supplemented with 10^-7^ M E2. Confluent layers were then wounded and cells were allowed to migrate for the indicated time. Shown is an experiment performed with cells from donor 1. **b.** Quantification of the wound healing assays. Areas not covered by cells were quantified and were expressed relative to 0h time point. Results are shown as the average of six independent experiments performed in triplicate with error bars representing s.e.m. Significance was analyzed using ANOVA tests. ns = not significant, ***: *p*<0.001
(TIF)

S3 Fig 17β-estradiol does not regulate collagen secretion and mRNA expression. **a.** hDF were cultured in the presence of untreated (FCS) or desteroidated serum (DS) for 2 days, then supplemented with 10^-7^ M E2 and ascorbic acid. Expression of secreted collagen I, V and VI was analyzed by western blot. Ponceau staining is shown on the lower panel. Shown are the results obtained with donor 4. Quantification of protein expression (displayed below the blots) is expressed relative to Ponceau staining (shown on the lower panel) with FCS condition assigned to 1 as mean±SEM n = 4 donors. **b.** Expression of the indicated mRNA analyzed by real-time PCR. Data are presented relative to vehicle treated samples and are the average of experiments performed twice on 4 donors in triplicate. Error bars indicate SEM. Variations are not significant as estimated by ANOVA tests.
(TIF)

S4 Fig Efficiency of ICI182,780 treatment in hDF cells. hDF cells were transfected with ERE-luc vector supplemented or not with ER-encoding plasmid and treated with the indicated compounds. Luciferase activities were determined and are expression relative to β-Gal activities brought by a co-transfected CMV-β-Gal plasmid. Shown are the results of three independent transfections each performed in two donors in triplicate with error bars indicating s.e.m. Significance was analysed using ANOVA tests. ***: *p*<0.005.
(TIF)

S5 Fig Effect of 17β-estradiol is independent from PI3K and EGFR. **a.** Expression of EGFR and GPR30 was determined in hDF and MDA-MB231 breast cancer cells by qPCR. Expressions are indicated relative to that of the 36b4 housekeeping gene (values are in Ct of the indicated genes minus that of 36b4). Experiments were performed on the four donors in triplicate. Values are mean+/-s.e.m.**b,c.** Cells from individual donors were cultured in DS medium for 2 days and treated with E2, supplemented as indicated with 10^-5^ M AG1478 (AG; EGFR inhibitor) or 10^-5^ M LY294002 (LY; PIK3 inhibitor) for 4h. **b.** Upper panel: expression of phosphorylated ERK (p-ERK), total ERK (t-ERK) and hsp90 in cells from donor 4 shown as illustration. Lower panel: quantification of the western blots performed on the four donors. Data are presented as mean±SEM relative to vehicle-treated control. **c.** Cells shape monitored under the indicated conditions as in Fig. 1b. n = 400 cells. ns: not significant; ***p<0.001.
(TIF)

S1 Video hDF cells videorecorded for 8 h under DS + E2 conditions. Cells were cultured in DS medium for 2 days and videorecorded when E2 was added.
(AVI)

S2 Video hDF cells videorecorded for 8h under DS conditions. Cells were cultured in DS medium for 2 days before being videorecorded.
(AVI)
