# Supplementary figures and images for: Estrogens Induce Rapid Cytoskeleton Re-Organization in Human Dermal Fibroblasts via the Non-Classical Receptor GPR30
Source: PLoS One. 2015 Mar 17;10(3):e0120672. doi: 10.1371/journal.pone.0120672 (PMC4363467; doi:10.1371/journal.pone.0120672)

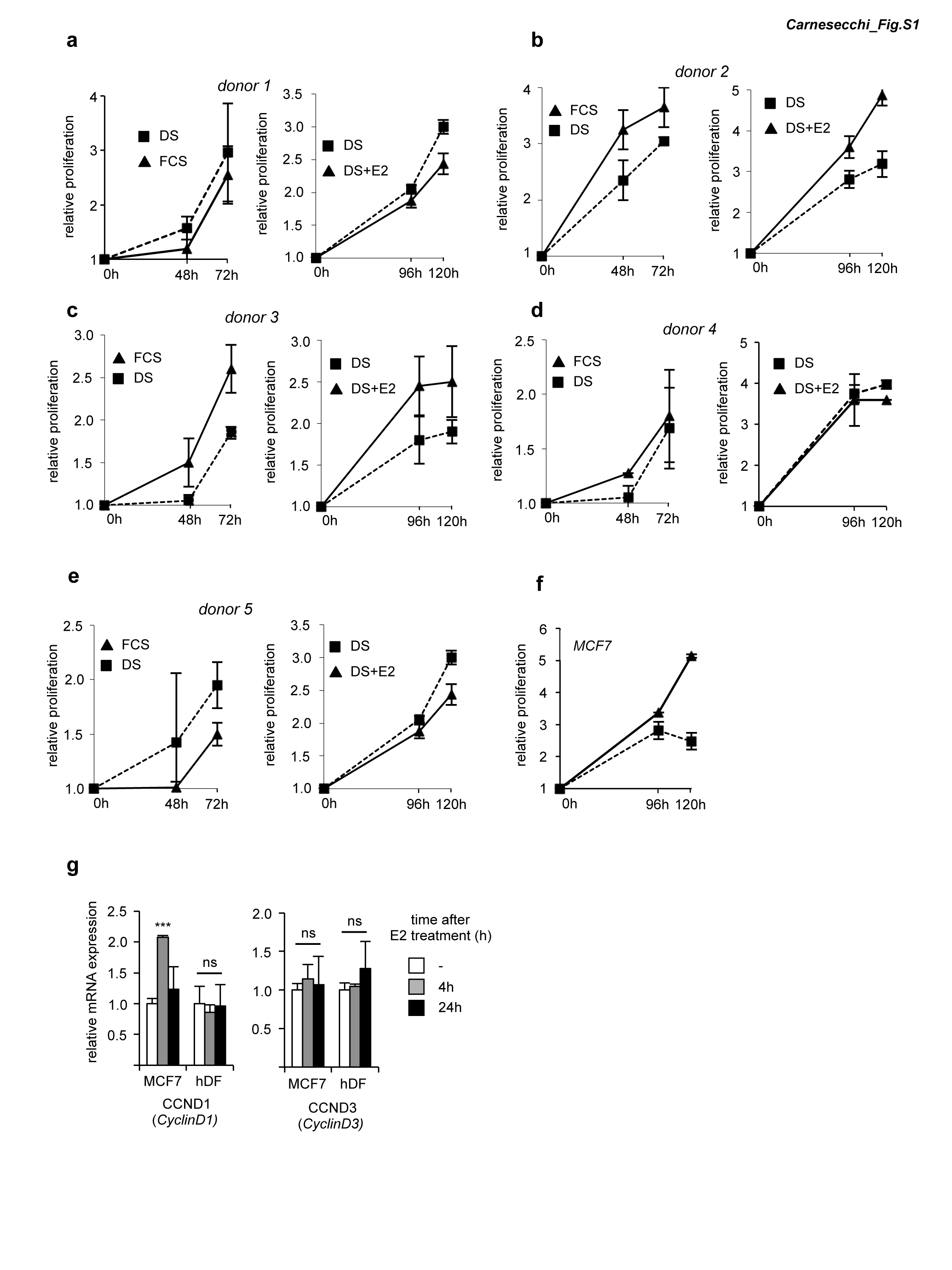

Supplement: S1 Fig — Cells from donors 1 to 5 (a to e, respectively) were cultured in the presence of untreated (FCS) vs desteroidated serum (DS) (left panels) or in DS-containing medium supplemented with vehicle (DS) or 10-7 M 17β-estradiol (DS+E2) (right panels). f. MCF7 cells were cultured in DS-containing medium supplemented or not with 10-7 M 17β-estradiol. Proliferation is expressed relative to 0h. Values represent a single experiment performed in duplicate on single donors (two experiments performed per donor with similar results) with error bar representing S.D. Significance was estimated using one way ANOVA test. ***: p<0.001. Donor 3 displayed a low proliferation rate and was excluded from further analyses. g. Expression of cyclin D1 or D3 (CCND1 and CCND3, respectively) in hDF or MCF7 cells under the indicated conditions, determined by qPCR. Results are expressed relative to the expression of the 36b4 housekeeping gene. Experiments were performed on each for donors in triplicate. Values are mean+/-s.e.m. ns = not significant, ***: p<0.001. (TIF) [file pone.0120672.s001.tif]

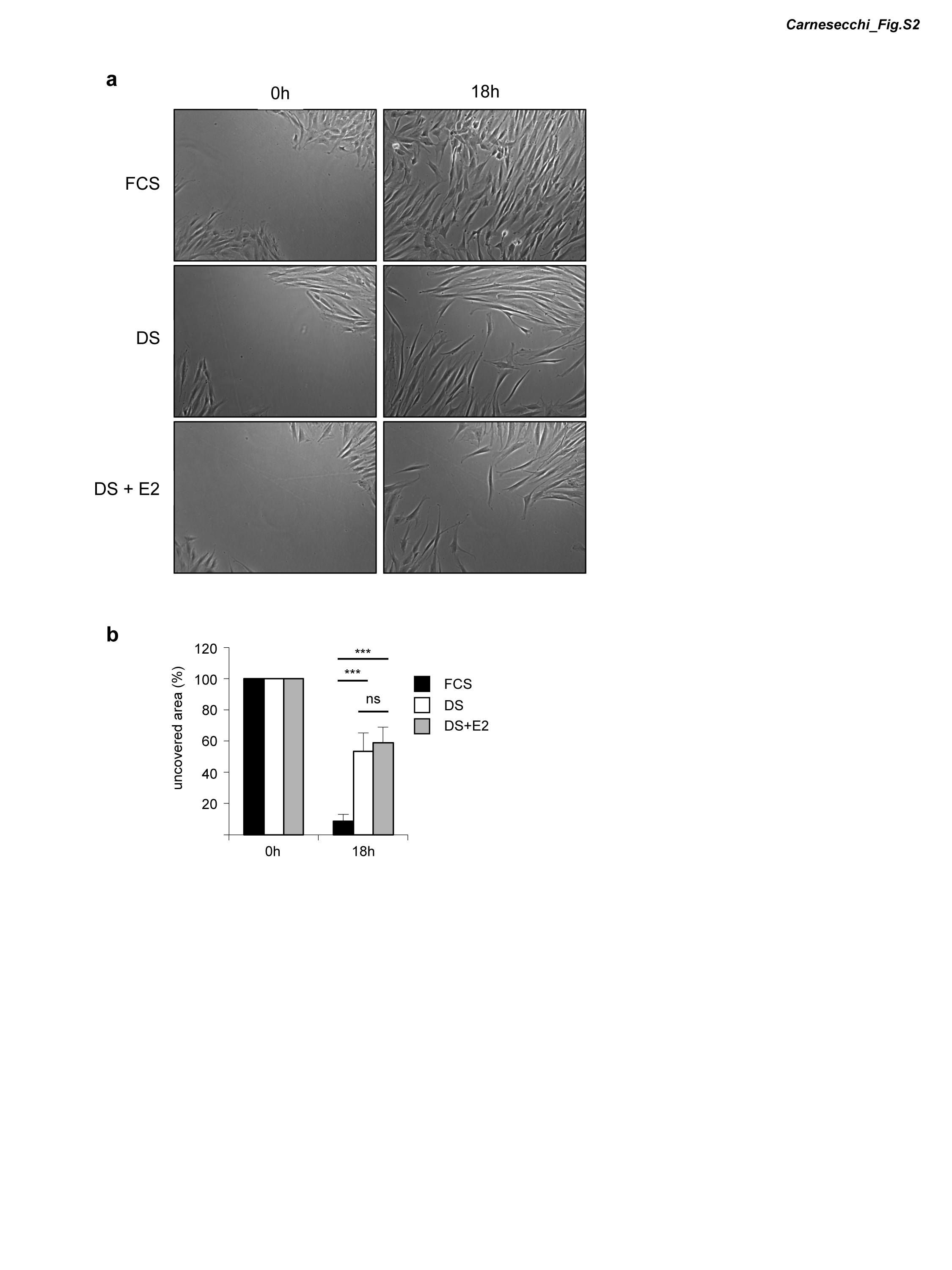

Supplement: S2 Fig — a. hDF were cultured in the presence of untreated (FCS) or desteroidated serum (DS) for 2 days, then supplemented with 10-7 M E2. Confluent layers were then wounded and cells were allowed to migrate for the indicated time. Shown is an experiment performed with cells from donor 1. b. Quantification of the wound healing assays. Areas not covered by cells were quantified and were expressed relative to 0h time point. Results are shown as the average of six independent experiments performed in triplicate with error bars representing s.e.m. Significance was analyzed using ANOVA tests. ns = not significant, ***: p<0.001 (TIF) [file pone.0120672.s002.tif]

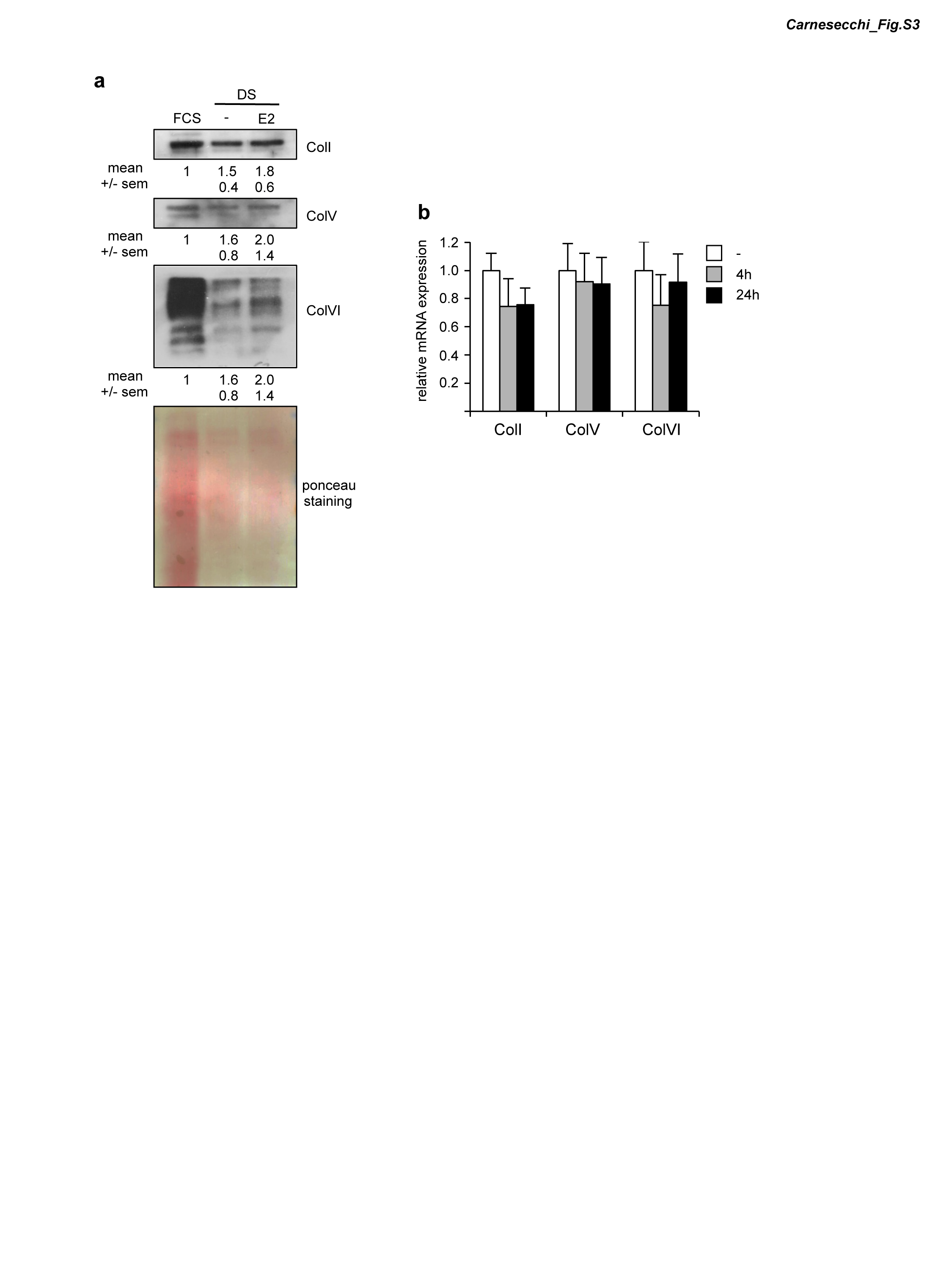

Supplement: S3 Fig — a. hDF were cultured in the presence of untreated (FCS) or desteroidated serum (DS) for 2 days, then supplemented with 10-7 M E2 and ascorbic acid. Expression of secreted collagen I, V and VI was analyzed by western blot. Ponceau staining is shown on the lower panel. Shown are the results obtained with donor 4. Quantification of protein expression (displayed below the blots) is expressed relative to Ponceau staining (shown on the lower panel) with FCS condition assigned to 1 as mean±SEM n = 4 donors. b. Expression of the indicated mRNA analyzed by real-time PCR. Data are presented relative to vehicle treated samples and are the average of experiments performed twice on 4 donors in triplicate. Error bars indicate SEM. Variations are not significant as estimated by ANOVA tests. (TIF) [file pone.0120672.s003.tif]

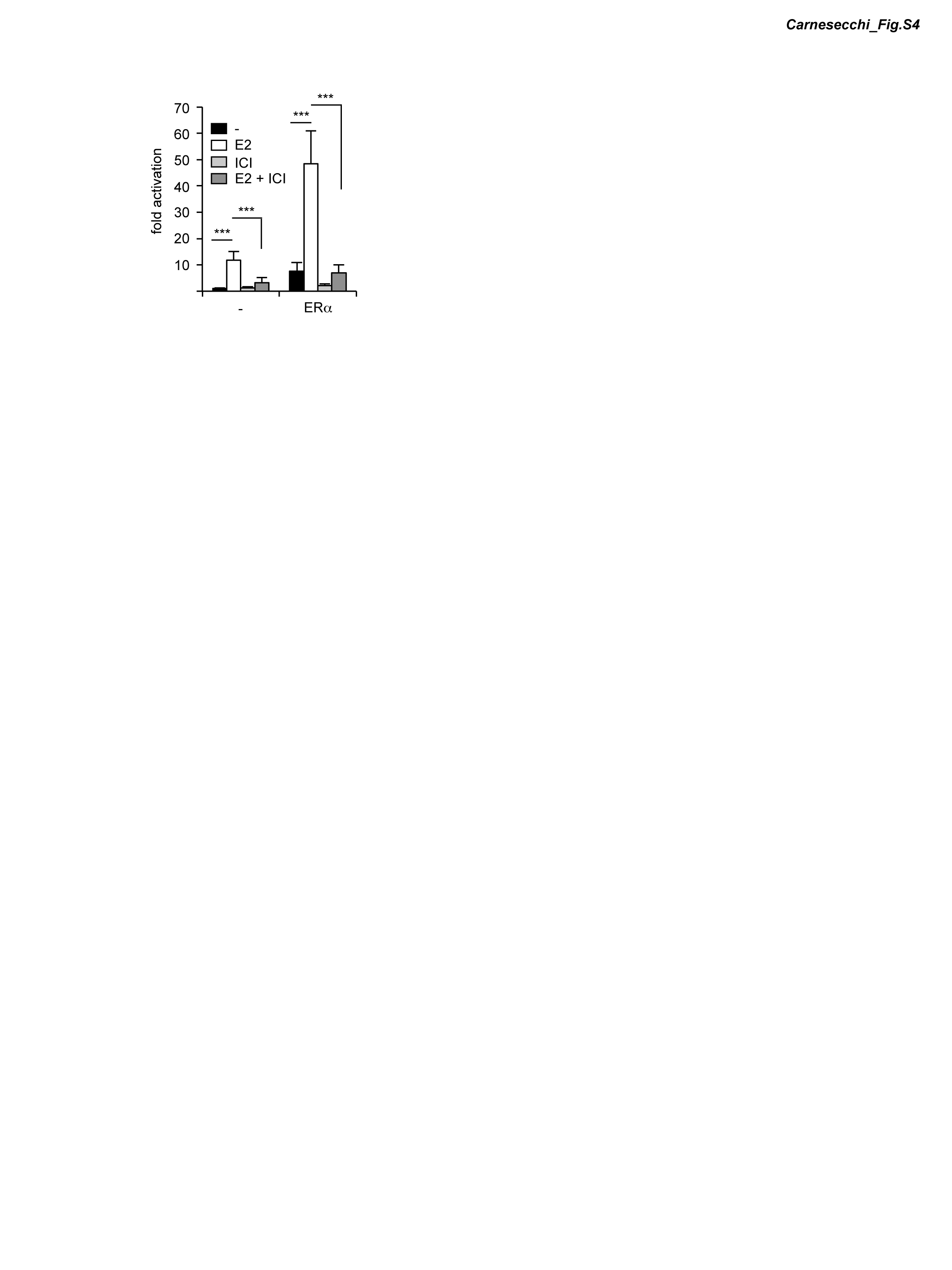

Supplement: S4 Fig — hDF cells were transfected with ERE-luc vector supplemented or not with ER-encoding plasmid and treated with the indicated compounds. Luciferase activities were determined and are expression relative to β-Gal activities brought by a co-transfected CMV-β-Gal plasmid. Shown are the results of three independent transfections each performed in two donors in triplicate with error bars indicating s.e.m. Significance was analysed using ANOVA tests. ***: p<0.005. (TIF) [file pone.0120672.s004.tif]

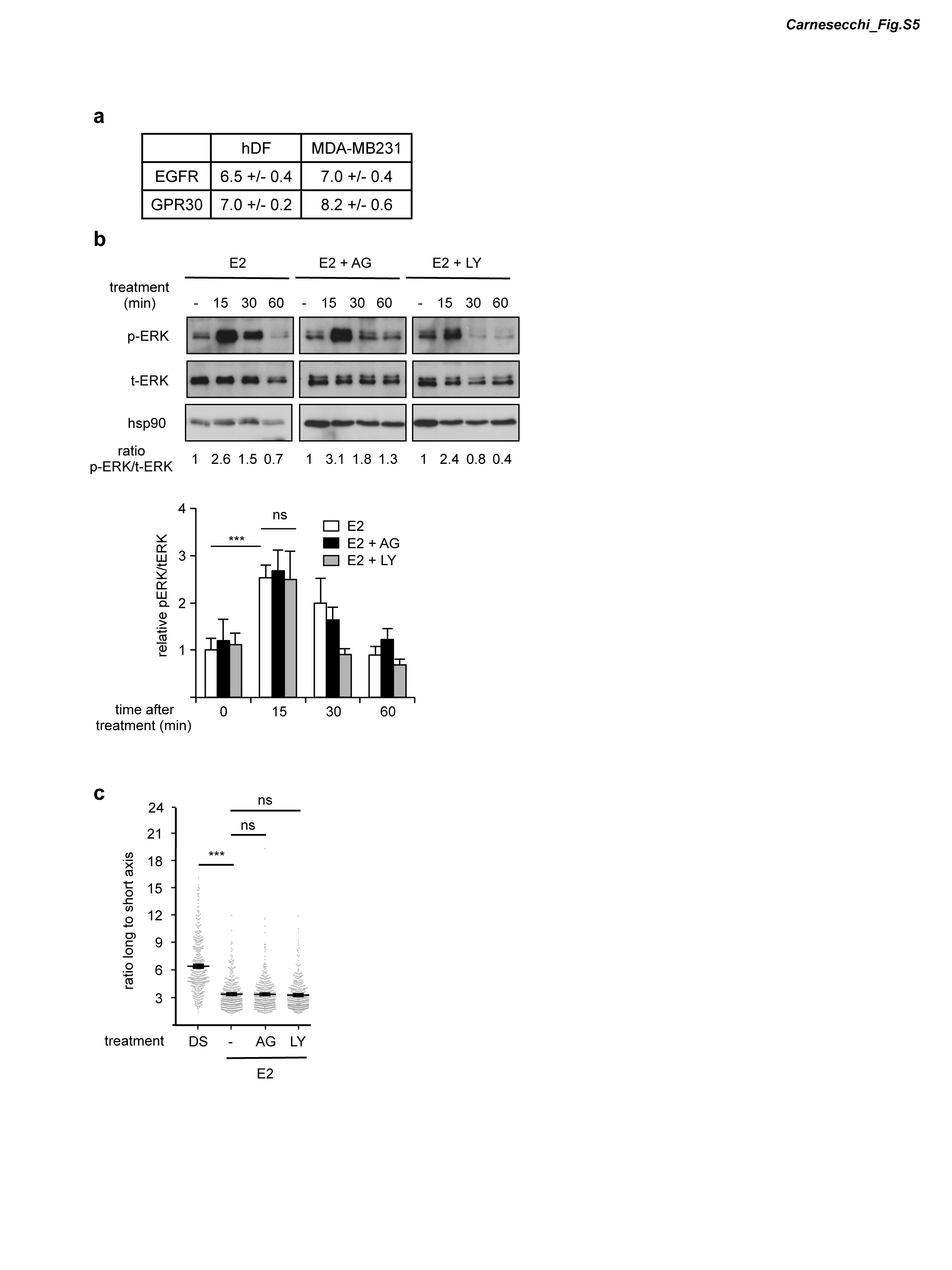

Supplement: S5 Fig — a. Expression of EGFR and GPR30 was determined in hDF and MDA-MB231 breast cancer cells by qPCR. Expressions are indicated relative to that of the 36b4 housekeeping gene (values are in Ct of the indicated genes minus that of 36b4). Experiments were performed on the four donors in triplicate. Values are mean+/-s.e.m.b,c. Cells from individual donors were cultured in DS medium for 2 days and treated with E2, supplemented as indicated with 10-5 M AG1478 (AG; EGFR inhibitor) or 10-5 M LY294002 (LY; PIK3 inhibitor) for 4h. b. Upper panel: expression of phosphorylated ERK (p-ERK), total ERK (t-ERK) and hsp90 in cells from donor 4 shown as illustration. Lower panel: quantification of the western blots performed on the four donors. Data are presented as mean±SEM relative to vehicle-treated control. c. Cells shape monitored under the indicated conditions as in Fig. 1b. n = 400 cells. ns: not significant; ***p<0.001. (TIF) [file pone.0120672.s005.tif]
